# Contrast-Induced Acute Kidney Injury in Patients with Heart Failure on Sodium–Glucose Cotransporter-2 Inhibitors Undergoing Radiocontrast Agent Invasive Procedures: A Propensity-Matched Analysis

**DOI:** 10.3390/jcm13072041

**Published:** 2024-04-01

**Authors:** Giulia Nardi, Enrico Marchi, Marco Allinovi, Gianmarco Lugli, Lucrezia Biagiotti, Francesca Maria Di Muro, Renato Valenti, Iacopo Muraca, Benedetta Tomberli, Niccolò Ciardetti, Brunetto Alterini, Francesco Meucci, Carlo Di Mario, Alessio Mattesini

**Affiliations:** 1 Department of Experimental and Clinical Medicine, School of Human Health Sciences, Careggi University Hospital, University of Florence, 50134 Florence, Italy; giulia.nardi.fi@gmail.com (G.N.); enrico.marchi1@gmail.com (E.M.); lucrezia.biagiotti@unifi.it (L.B.); francescamaria.dimuro@unifi.it (F.M.D.M.); carlo.dimario@unifi.it (C.D.M.); 2 Nephrology, Dialysis and Transplantation Unit, Careggi University Hospital, 50134 Florence, Italy; 3 Division of Interventional Cardiology, Cardiothoracovascular Department, Careggi University Hospital, 50134 Florence, Italy; valentir@aou-careggi.toscana.it (R.V.); muracai@aou-careggi.toscana.it (I.M.); 4 Division of General Cardiology, Cardiothoracovascular Department, Careggi University Hospital, 50134 Florence, Italy; tomberlib@aou-careggi.toscana.it; 5 Division of Structural Interventional Cardiology, Cardiothoracovascular Department, Careggi University Hospital, 50134 Florence, Italy; niccolo.ciardetti@unifi.it (N.C.); meuccif@aou-careggi.toscana.it (F.M.); mattesinia@aou-careggi.toscana.it (A.M.); 6 Division of Cardiovascular and Perioperative Medicine, Cardiothoracovascular Department, Careggi University Hospital, 50134 Florence, Italy; alterinib@aou-careggi.toscana.it

**Keywords:** contrast-induced acute kidney injury, SGLT2-inhibitors, gliflozins, heart failure, nephrotoxicity

## Abstract

(1) **Background**: This single-center retrospective study aimed to evaluate whether sodium–glucose cotransporter-2 inhibitors (SGLT2-i) therapy may have a nephroprotective effect to prevent contrast-induced acute kidney injury (CI-AKI) in patients with heart failure (HF) undergoing iodinated contrast medium (ICM) invasive procedures. (2) **Methods**: The population was stratified into SGLT2-i users and SGLT2-i non-users according to the chronic treatment with gliflozins. The primary endpoint was CI-AKI incidence during hospitalization. Secondary endpoints were all-cause mortality and the need for continuous renal replacement therapy (CRRT). (3) **Results**: In total, 86 patients on SGLT2-i and 179 patients not on SGLT2-i were enrolled. The incidence of CI-AKI in the gliflozin group was lower than in the non-user group (9.3 vs. 27.3%, *p* < 0.001), and these results were confirmed after propensity matching analysis. Multivariable logistic regression showed that only SGLT2-i treatment was an independent preventive factor for CI-AKI (OR: 0.41, 95% CI: 0.16–0.90, *p* = 0.045). The need for CRRT was reported only in five patients in the non-SGLT2-i-user group compared to zero patients in the gliflozin group (*p* = 0.05). (4) **Conclusions**: SGLT2-i therapy was associated with a lower risk of CI-AKI in patients with HF undergoing ICM invasive procedures.

## 1. Introduction

Heart failure (HF) is a clinical syndrome with high prevalence in Western countries and a major impact on healthcare systems [1]. Progressive worsening of renal function is part of the natural history of HF since almost half of patients with HF have developed renal impairment [2]. This condition is often accelerated by nephrotoxic damage related to the use of iodinated contrast medium (ICM) for coronary examinations and repeated percutaneous coronary intervention (PCI).

It is widely established that HF is a well-recognized risk factor for contrast-induced acute kidney injury (CI-AKI) [3]. Although there is clear evidence that CI-AKI has an impact on mortality [4,5], there are no drug therapies that can significantly mitigate this risk to date [6]. On the contrary, it is known from randomized clinical trials that sodium–glucose cotransporter-2 inhibitors (SGLT2-i) have a nephroprotective effect with a reduction in renal disease mortality [7]. There is weak but consistent evidence for the protection from CI-AKI conferred by gliflozin therapy. Available data include retrospective registries of patients with diabetes, acute coronary syndrome or chronic ischemic heart disease undergoing diagnostic or invasive coronary examinations with ICM [8]. In these patient groups, gliflozin treatment started before and continued after ICM administration reduced the likelihood of developing CI-AKI. Since patients with HF have a very high risk of CI-AKI, the expected benefit of gliflozin treatment is even higher, but the level of evidence is poor in this setting. We therefore conducted this retrospective study to evaluate the potential nephroprotective effect of SGLT2-i therapy to prevent CI-AKI in patients with HF undergoing invasive procedures with ICM.

## 2. Materials and Methods

### 2.1. Study Population

This single-center, retrospective study enrolled all consecutive patients with HF undergoing ICM invasive procedures at Careggi University Hospital from January 2019 to December 2023, including both coronary and structural Interventions.

Automatic software-based research was performed through electronic medical records and operative logs for all consecutive patients who underwent coronary angiography, PCI, transcatheter aortic valve replacement or another iodinated contrast medium procedure. Among these, we identified those with HF and reduced or mildly reduced ejection fraction (HFrEF or HFmrEF) and, based on HF therapy at the admission, we categorized them into SGLT2-i users (in the era of gliflozins prescribed for HF) and SGLT2-i non-users (in the pre-gliflozin era).

Definition of HF with reduced left ventricular ejection fraction (LVEF) (HFrEF, LVEF ≤ 40%) and mildly reduced LV systolic function (HFmrEF, LVEF between 41% and 49%) was based on the most recent ESC guidelines [9]. Patients with HF and preserved LVEF (HFpEF) were excluded.

We defined “SGLT2-i users” the patients who were admitted on chronic SGLT2-i therapy (empagliflozin or dapagliflozin) and started it at least 6 months before PCI. This cut-off of 6 months was chosen since SGLT2-i may exhibit beneficial effects after a 6-month period, as indicated in several studies [10,11].

Indication for invasive procedures (coronary angiography, PCI or structural interventions, patent foramen oval closure and left atrial appendage occlusion, or endomyocardial biopsy), referral, and timing were managed according to the current guidelines and after a multidisciplinary discussion by a team of expert in coronary and valvular heart disease, including a clinical and interventional cardiologist, cardiac surgeons, imaging specialists with expertise in interventional imaging, cardiovascular anesthesiologists, and other specialists if necessary (e.g., heart failure specialists or electrophysiologists)—the “Heart Team”.

The exclusion criteria were as follows: cardiogenic shock and multi-organ failure or hemodynamic instability requiring ino-vasopressors agents, dialysis treatment or continuous renal replacement therapy (CRRT) at the time of the procedure, patients on SGLT2-i therapy for less than 6 months, and incomplete information on medical therapy.

All patients were informed about their participation in the study and provided informed consent for the anonymous publication of scientific data.

### 2.2. Endpoints

The primary endpoint was the incidence of CI-AKI during the hospitalization. Serum creatinine concentration and estimated glomerular filtration rate (eGFR) according to the Chronic Kidney Disease Epidemiology Collaboration (CKD-EPI) equation were measured in all patients at hospital admission and daily during the hospital stay. CI-AKI was defined as an absolute (≥0.5 mg/dL) or relative increase (≥25%) in serum creatinine at 48–72 h after the index PCI compared to baseline serum creatinine values according to the latest definition [12]. CI-AKI was considered either as a new-onset or an exacerbation of renal dysfunction following administration of ICM without other potential causes. We considered the Mehran score, which includes the most clinically relevant variables associated with CI-AKI, i.e., age, anemia, contrast media volume, eGFR, congestive heart failure, hypotension, use of intra-aortic balloon pump to predict the risk of CI-AKI after PCI [13].

To prevent contrast-induced nephropathy, according to our institutional protocol, all patients in the study underwent pre-procedural intravenous fluid administration of 1 mg/kg/h normal saline or Hartmann’s solution if in the HFmrEF group or 0.5 mg/kg/h if in the HFrEF group.

The iodinated contrast available in our institution is Iobitridol 350 (Xenetix^®^, Guerbet, Villepinte, France), which was used in all patients undergoing coronary angiography and other procedures in our institution.

The secondary endpoint was a composite of all-cause mortality at median follow-up and need for in-hospital hemodialysis, also including CRRT. We considered both patients who underwent in-hospital CRRT after the ICM procedure and those who underwent prophylactic CRRT by receiving hemodialysis either before or after the administration of radiocontrast agent in case of pre-existing renal failure, since in the literature, the evidence regarding the role of CRRT in CI-AKI prevention is controversial [14].

### 2.3. Statistical Analysis

Histograms and q-plot assessed the normal distribution of continuous variables; the Shapiro–Wilk test was used when required. Continuous variables with normal distribution were expressed as the mean ± standard deviation and non-normally distributed as median and interquartile range. Normal ranges were presented as the 5th and 95th percentiles. Categorical variables were expressed as counts and percentages. Differences between groups were analyzed using the *t*-test or the Mann–Whitney U test for continuous variables and the chi-square test or the Fisher’s exact test for categorical variables, as appropriate.

Propensity scores were calculated using logistic regression of SLGT2 inhibitor use on age, gender, body mass index (BMI), hypertension, heart failure class, LVEF at admission, eGFR at admission, prevalent peripheral arterial disease, prevalent diabetes, prevalent atrial fibrillation, and smoking. For participants with missing values in any of these covariates, exact matches were required on missing status. Propensity matching was performed with a 1:1 match for case and control subjects in which the nearest neighbor was selected without replacement.

A *p*-value < 0.05 was considered statistically significant. All analyses were performed using Statistical Package for Social Sciences, version 28.0 (SPSS, Chicago, IL, USA) and R version 4.0.

Further details regarding the propensity matching analysis are reported in Figures S1–S3 available in the Supplementary Materials.

## 3. Results

### 3.1. Baseline Characteristics and Propensity Score

We enrolled a total of 265 patients with HFrEF undergoing ICM invasive procedures at the Careggi University Hospital from January 2019 to December 2023.

Among these, we identified 86 patients with HF on SGLT2-i therapy and 179 patients with HF and not on SGLT2-i who underwent radiocontrast agent invasive procedures before matching.

**Figure 1:**
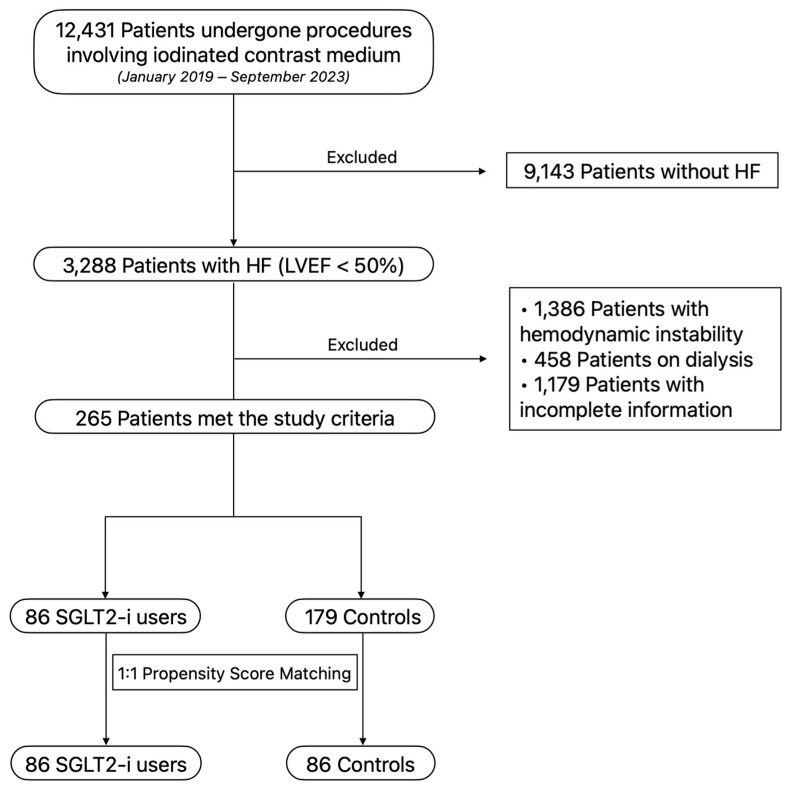
Flowchart of study population to outline the selection of unmatched and propensity score-matched patients of our study.

The flowchart of our study population to outline the selection of unmatched and propensity score-matched patients is represented in Figure 1.

Compared with non-users, SGLT2-i users were younger and had a higher prevalence of diabetes and dyslipidemia, a history of peripheral artery disease, previous myocardial infarction, and previous PCI, and they had a shorter hospital stay (Table 1). Furthermore, considering HF therapies, SGLT2-i users had a more frequent prescription of renin–angiotensin–aldosterone system inhibitors (RAAS-i), statins and diuretics (including both loop and thiazides) at the admission. LVEF was similar between the two groups, with a mean value of 36% (*p* = 0.5). Median NT-proBNP levels were 1337 pg/mL in the treatment group and 2883 pg/mL in the non-user group (*p* = 0.003). Other baseline population characteristics before propensity matching are reported in Table 1.

SGLT2-i users also had higher serum creatinine levels at admission (*p* = 0.04). A higher proportion of metformin usage and Angiotensin-Receptor Neprilysin Inhibitor (ARNI) were observed in the SGLT2-i users than non-users.

Most of the SGLT2-i users were on dapagliflozin 10 mg (65%), followed by empagliflozin 10 mg (20%), and then dapagliflozin 25 mg (15%). In the SGLT2-i group, the incidence of CI-AKI was significantly lower than the non-user group (9.3% vs. 27.3%, *p* < 0.001). Five patients (2.8%) in the non-user group underwent CRRT during the hospitalization and all-cause mortality was higher in SGLT2-i non-users compared to users (15.1% vs. 3.5%, respectively, *p* = 0.005).

After propensity score matching, we identified 86 SGLT2-i non-users matched to the 86 SGLT2-i users previously identified (Table 2). Users and non-users were well matched except for PCI and acute myocardial infarction (AMI) history (50.0% vs. 26.4% in users vs. non-users), diagnosis at admission (7.0% vs. 19.8% ST-elevated myocardial infarction (STEMI) in users vs. non-users), dyslipidemia (87.2% vs. 54.0%) and RAAS-i, statin and diuretics usage (76.7%–84.9%–68.6% vs. 53.2%–45.3%–44.1% in users vs. non-users) in the group (Table 2).

### 3.2. Primary Endpoint

After propensity matching, the proportion of patients with CI-AKI events in SGLT2-i users and non-users was 9.3 and 26.7%, respectively (*p* = 0.016). The occurrence of acute dialysis or the need for CRRT due to contrast-induced acute kidney injury was observed in none of the patients in the user group, whereas it was reported in five patients in the non-user group (*p* = 0.059).

The median follow-up was 13.6 ± 8.4 months in the SGLT2-i users, while on the contrary was 49.9 ± 13.6 months in the non-user group after the index procedure with contrast medium.

The univariable logistic regression analysis showed that only SGLT2-i therapy (OR: 0.36, 95% CI: 0.15–0.82, *p* = 0.019) was an independent protective factor of CI-AKI for patients undergoing PCI, and this result was also confirmed by the multivariable logistic regression analysis (OR: 0.41, 95% CI: 0.16–0.90, *p* = 0.045) (Table 3).

### 3.3. Secondary Endpoints

Regarding the secondary endpoints, a composite of renal failure requiring in-hospital CRRT and all-cause mortality, eGFR at the admission and the Mehran score were independent predictors at the univariable and multivariable analysis (OR: 0.95, 95% CI: 0.92–0.98, *p* < 0.001; OR: 1.28, 95% CI: 1.13–1.47, *p* < 0.001, respectively), whereas SGLT2-i assumption was confirmed as a protective factor (OR: 0.12, 95% CI: 0.03–0.40, *p* = 0.002) (Table 4).

**Figure 2:**
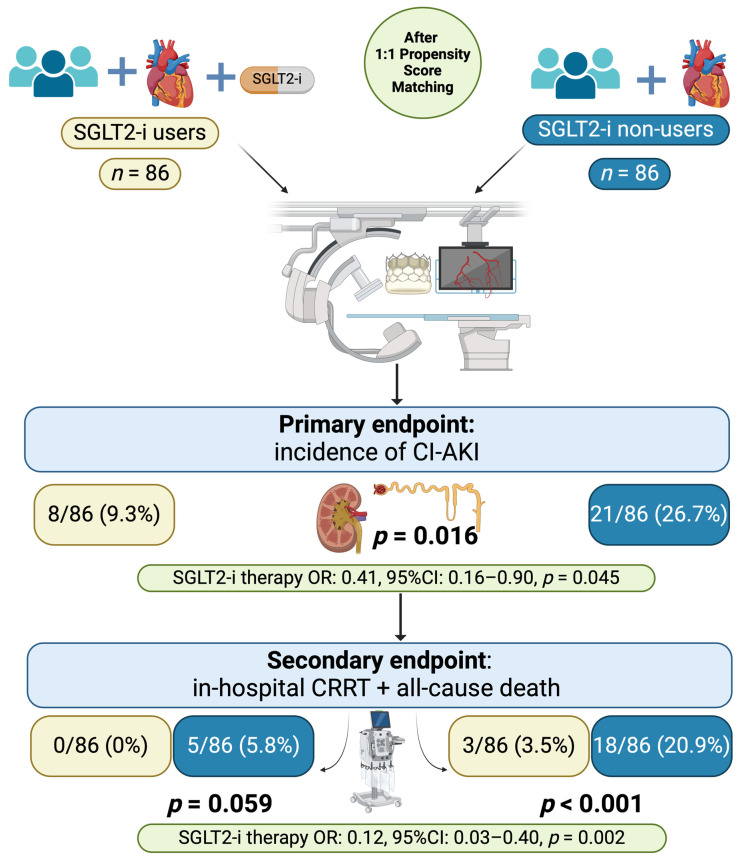
Study result illustration after propensity matching analysis with primary and secondary endpoints summarized.

All these results are illustrated in Figure 2 in which the primary endpoint of incidence of CI-AKI and the secondary composite endpoint of all-cause death and need for in-hospital mortality are accurately reported and OR and CI for each predictive factor are underlined.

## 4. Discussion

In the present study, we investigated the potential impact of SGLT2-i in reducing the risk of CI-AKI among HF patients. The most important result was a clear association between SGLT2i treatment and a reduced risk of CI-AKI. This finding was persistent after propensity matching analysis and correction for potential confounding by multivariable analysis. A reduction in the composite secondary endpoint (renal failure requiring CRRT + all-cause mortality) was also observed.

SGLT2-i exhibited a consistent benefit of the reduction in CI-AKI across different patients’ comorbidity and indications to undergo invasive procedures with ICM suggesting its protection in all spectra of HF patients, with no differences based on the presence of pre-existing chronic kidney disease and/or diabetes, as already demonstrated in previous investigations [15]. SGLT2-i use was also associated with mortality reduction probably due to the reno-protective effect, since the post-procedural need for CRRT was lower among patients treated with gliflozins.

To the best of our knowledge, this is the first study on the potential protective role of SGLT2-i from CI-AKI in HF patients undergoing invasive procedures with ICM, independently from the presence of diabetes, chronic kidney disease and acute coronary syndromes.

In the literature, some data suggesting a protective role of SGLT2-i from CI-AKI are available, but only in specific settings such as diabetes, chronic kidney disease (CKD) patients and patients with AMI undergoing primary PCI.

Despite an initial warning advanced by the Food and Drug Administration about the risk of AKI related to the use of gliflozins [16], further studies aimed to demonstrate that this class of drugs not only do not increase the risk of AKI but also presents a nephroprotective effect associated with CI-AKI prevention compared to other glucose-lowering therapies [17,18].

Firstly, Hua et al. [19] demonstrated the safety of SGLT2-i treatment administered at least 6 months before and continued after PCI. In this study, the authors not only did not observe any increased risk of CI-AKI with SGLT2-i usage after PCI but also observed an unadjusted ORs of CI-AKI KDIGO 54% lower in the SGLT2-i user group compared with the non-user group [0.46 (95% CI: 0.276–0.75); *p* = 0.02]. This study was clinically relevant as it provided the first evidence regarding the safety of gliflozin treatment without the need for discontinuation prior to exposure to contrast media. Similar safety data are not definitive for other classes of glucose-lowering drugs such as metformin.

With these premises, other studies investigated the role of SGLT2-i for renal protection in specific populations undergoing ICM procedures. In a large cohort of type 2 diabetes mellitus (T2DM) patients, a propensity-matched analysis published by Nadkarni et al. [20] showed that the CI-AKI risk was reduced with SGLT2-i treatment (HR 0.4 [95% CI: 0.2–0.7]; *p* = 0.01).

Furthermore, in a multicentre international registry (the SGLT2-i AMI PROTECT registry) recently published by Paolisso et al. [8], the authors demonstrated that the SGLT2-i use was associated with a lower risk of CI-AKI in T2DM patients with AMI, mostly in patients without CKD. However, as mentioned before, this study included only diabetic patients with AMI. We should acknowledge that patients with AMI have a higher risk of developing renal failure due to the high thrombogenic state, the inflammation status, and the decrease in renal perfusion. On the contrary, our population included a large proportion of patients with CKD and chronic coronary syndromes.

Well-established risk factors for CI-AKI included age, diabetes, CKD, HF, and other elements related to intervention procedures, such as type and amount of ICM usage, repeated PCI, and use of mechanical circulatory support such as IABP, as assessed by the Mehran score. In addition to what is reported in the literature, our multivariable regression analysis showed that SGLT2-i usage was a predictor of CI-AKI (Table 3).

Furthermore, SGLT2-i use was also an independent protective factor for the secondary composite endpoint of renal failure requiring CRRT and/or all-cause mortality, whereas independent related risk factors were eGFR at admission and the Mehran score (Table 4). These results are congruent with previous studies reporting the prognostic significance of the proposed score to predict one-year mortality and the need for in-hospital hemodialysis [13].

All the above-mentioned studies probably share a common beneficial effect conferred by gliflozins therapy. In fact, although the underlying mechanisms are not completely understood, the nephroprotective effect of SGLT2-i appears to be independent of the blood-glucose-lowering effects. Actually, gliflozin’s effects might be mediated by natriuresis and glucose-induced osmotic diuresis, leading to a reduction in intraglomerular pressure (and consequently a reduction in hyperfiltration) and exposure time of each nephron to contrast agent direct toxicity and a decrease in ischemic injury mediated by vasoconstriction and limited renal blood flow [21]. Therefore, gliflozins are involved in maintaining the glomerular balance between renal perfusion, filtration rate and fluid reabsorption in the proximal tubule, a vulnerable system that is perturbated in HF. In addition, in patients with HF, the renal hemodynamic effects of the SGLT2-i resulting from pre-glomerular vasoconstriction is often combined with the efferent arteriolar vasodilatation mediated by RAAS inhibitors, another pillar of HF therapy. Thus, a combination of SGLT2-i and RAAS inhibitors may result in a nephroprotective synergic therapy and CI-AKI prevention strategy in HF patients.

Emerging data from Huang X et al. [22] demonstrated that even dapagliflozin may ameliorate CI-AKI through suppression of HIF-1a/HE4/NF-kB signaling in vitro and in vivo, which is the pathway involved in hypoxia-induced injury. Furthermore, SGLT2-i can affect vascular remodeling and neointimal hyperplasia after ICM [7]. Other renal protective effects could be represented by inflammatory response modulation, reduction in endothelial activation, fibrosis and oxidative stress production [23].

In our experience, gliflozin use was also associated with a significative lower mortality, and this result was maintained even after propensity-matched analysis. This result is also consistent with mortality data derived from pivotal trials about gliflozins in the HF population [7,11,24].

The results of our study provide further evidence of the potential role of gliflozins in CI-AKI prevention. To date, no drugs have been demonstrated to have this role. There is only some evidence of statins providing renal protection when administered before ICM [25]. The only ascertained preventive strategies remain adequate volume expansion and reduction in the contrast dose administered [26]. In the future, randomized clinical trials are needed to definitively clarify the role of SGLT2-i as a key element for CI-AKI prevention, with tailored protocols of administration before and after ICM procedures, especially in CI-AKI high-risk patients.

Our study presents some limitations. First, the major limitation is the retrospective nature of the study with the two groups of patients enrolled in two different periods: pre- and post-SGLT2 prescription and reimbursement for HF, even though we conducted propensity score-matched analysis to minimize the impact of confounding factors

Second, even if the multivariable analysis clearly showed that contrast medium volume was not associated with a higher risk of CI-AKI, we should highlight that patients in the non-user group were exposed to a larger volume of ICM.

Thus, a larger sample size and multicenter studies are needed to further confirm the effectiveness of gliflozins in reducing CI-AKI risk for patients scheduled for an ICM invasive procedure. Furthermore, the limited number of patients enrolled, and the short follow-up data are not sufficient to assess hard clinical endpoints, such as permanent replacement therapy, dialysis, and mortality, and to ascertain the impact of SGLT2-i on patients’ long-term prognosis.

## 5. Conclusions

In this retrospective single-center study, SGLT2-i use was an independent protective factor for the occurrence of CI-AKI in patients with HF undergoing contrast agent invasive procedures. The nephroprotective effect of SGLT2-i is maintained in patients with reduced and mildly reduced heart failure and is independent of the presence of T2DM.

## Figures and Tables

**Table 1:** Characteristics of patients in two groups before propensity matching.

Variables	SGLT2-i Users (*n* = 86)	SGLT2-i Non-Users (*n* = 179)	*p*-Value
Female, *n* (%)	12 (13.9)	55 (30.7)	0.003
Age, years (mean ± SD)	70.9 ± 8.9	74.3 ± 11.0	0.002
Smoking (active or past), *n* (%)	55 (64.0)	92 (51.4)	0.012
Diabetes, *n* (%)	49 (57.0)	53 (29.6)	<0.001
Hypertension, *n* (%)	65 (75.6)	136 (76.0)	0.8
Dyslipidemia, *n* (%)	75 (87.2)	99 (55.3)	<0.001
Obesity, *n* (%)	14 (16.2)	25 (13.9)	0.6
PAD, *n* (%)	17 (19.8)	18 (10.1)	0.029
Atrial fibrillation, *n* (%)	25 (29.1)	38 (21.2)	0.1
Previous PCI, *n* (%)	51 (59.3)	48 (26.8)	<0.001
Previous CABG, *n* (%)	10 (11.6)	15 (8.4)	0.4
Previous MI, *n* (%)	43 (50.0)	39 (21.9)	<0.001
Diagnosis			
NSTEMI, *n* (%)	9 (10.4)	45 (25.2)	
Unstable Angina, *n* (%)	7 (8.1)	9 (5)	
STEMI, *n* (%)	6 (7)	46 (25.7)	
Chronic coronary syndrome, *n* (%)	29 (33.7)	21 (11.8)	
Acute heart failure, *n* (%)	1 (1.2)	11 (6.1)	
Chronic heart failure, *n* (%)	34 (39.6)	47 (26.2)	
Physiologic variables			
BMI, kg/m^2^ (mean ± SD)	26.1 ± 4.3	25.9 ± 4.1	0.5
LVEF, % (mean ± SD)	36.2 ± 7.6	35.5 ± 8.1	0.5
Mehran score, (mean ± SD)	6.4 ± 4.1	6.8 ± 4.6	0.6
HF class			0.4
HFrEF, *n* (%)	48 (55.8)	110 (61.5)	
HFmrEF, *n* (%)	38 (44.2)	69 (38.5)	
Laboratory variables			
Creatinine admission, mg/dL (mean ± SD)	1.3 ± 0.5	1.1 ± 0.7	0.04
Hemoglobin admission, g/dL (mean ± SD)	13.3 ± 1.9	13.2 ± 2.1	0.5
HbA1c, mmol/mol (mean ± SD)	47.3 ± 11.2	47.1 ± 11.2	0.8
hsTnT peak, pg/mL (mean ± SD)	125.0 ± 262.1	189.1 ± 1049.7	0.035
eGFR, mL/min (mean ± SD)	58.3 ± 25.5	64.4 ± 58.3	0.7
NT-proBNP, pg/mL (median, IQR)	1337, 597.5–3449.8	2883, 860.5–9132.5	0.003
Medications			
SGLT2-i			
Dapaglifozin 5 mg, *n* (%)	0	0	
Dapaglifozin 10 mg, *n* (%)	56 (65.1)	0	
Empaglifozin 10 mg, *n* (%)	17 (19.8)	0	
Empaglifozin 25 mg, *n* (%)	13 (15.1)	0	
RAAS-i			<0.001
None, *n* (%)	20 (23.3)	73 (40.8)	
ACE-i, *n* (%)	18 (20.9)	74 (41.3)	
ARBs, *n* (%)	19 (22.1)	30 (16.8)	
ARNI, *n* (%)	29 (33.7)	2 (1.1)	
Statin			<0.001
None, *n* (%)	14 (16.3)	103 (57.5)	
High-intensity statin, *n* (%)	44 (51.2)	40 (22.3)	
Low-intensity statin, *n* (%)	29 (33.7)	36 (20.1)	
Diuretics			<0.001
None, *n* (%)	27 (31.4)	114 (63.7)	
Loop diuretics, *n* (%)	19 (22.1)	36 (20.1)	
Loop diuretics + MRA, *n* (%)	16 (18.6)	12 (6.7)	
MRA, *n* (%)	24 (27.9)	9 (5.0)	
Thiazide, *n* (%)	0	8 (4.5)	
Other type 2 diabetes mellitus therapies			0.006
None, *n* (%)	47 (54.7)	133 (74.3)	
Metformin, *n* (%)	21 (23.3)	28 (15.6)	
Sulfonylureas, *n* (%)	0 (0)	1 (0.6)	
DPP-4 inhibitors, *n* (%)	1 (1.2)	0 (0)	
GLP-1 receptor agonist, *n* (%)	2 (2.3)	0 (0)	
Insulin, *n* (%)	9 (10.5)	8 (4.5)	
Metformin + insulin, *n* (%)	4 (4.7)	8 (4.5)	
Other, *n* (%)	2 (2.3)	1 (0.6)	
Procedural variables			
PCI, *n* (%)	36 (41.9)	123 (68.7)	<0.001
Radial access, *n* (%)	65 (75.6)	99 (55.3)	0.003
Complex PCI, *n* (%)	12 (14.0)	39 (21.8)	0.13
IABP, *n* (%)	1 (1.2)	7 (3.9)	0.5
Contrast volume, mL (mean ± SD)	82.6 ± 53.8	124.2 ± 73.5	<0.001
Creatinine peak post, mg/dL (mean ± SD)	1.2 ± 0.5	1.3 ± 0.8	0.4
Outcomes			
CI-AKI, *n* (%)	8 (9.3)	49 (27.3)	<0.001
CRRT, *n* (%)	0	5 (2.8)	0.1
All-cause death, *n* (%)	3 (3.5)	27 (15.1)	0.005
Follow-up duration, months (mean ± SD)	13.6 ± 8.4	49.9 ± 13.6	<0.001

Continuous variables are presented as median (IQR) or mean ± D, whereas categorical variables are presented as *n* (%). ACE-i, angiotensin-converting enzyme inhibitors; ARBs, angiotensin receptor blockers; ARNI, Angiotensin Receptor Neprilysin Inhibitor; BMI, body mass index; CABG, Coronary Artery Bypass Graft surgery; CRRT, continuous renal replacement therapy; DPP-4 inhibitors, dipeptidyl peptidase 4 inhibitors; eGFR, estimated glomerular filtration rate; HbA1c, hemoglobin A1c; HF, heart failure; HFmrEF, heart failure with mildly reduced ejection fraction; HFrEF, heart failure with reduced ejection fraction; hsTnT, high sensitivity troponin T; IABP, Intra-Aortic Balloon Pump; LVEF, left ventricle ejection fraction; MI, myocardial infarction; NSTEMI, non-ST-elevated myocardial infarction; NT-proBNP, N-terminal pro-B-type natriuretic peptide; PAD, peripheral artery disease; PCI, percutaneous coronary intervention; RAAS-I, renin–angiotensin aldosterone system inhibitors; SGLT2-i, sodium–glucose cotransporter-2 inhibitors; STEMI, ST-elevated myocardial infarction.

**Table 2:** Characteristics of patients in two groups in propensity-matched dataset.

Variables	SGLT2-i Users (*n* = 86)	SGLT2-i Non-Users (*n* = 86)	*p*-Value
Female, *n* (%)	12 (13.9)	12 (14.0)	0.9
Age, years (mean ± SD)	70.9 ± 8.9	72.0 ± 12	0.2
Smoking (active or past), *n* (%)	55 (64.0)	51 (58.0)	0.15
Diabetes, *n* (%)	49 (57.0)	50 (58.1)	0.3
Hypertension, *n* (%)	65 (75.6)	62 (71.2)	0.6
Dyslipidemia, *n* (%)	75 (87.2)	47 (54.0)	<0.001
Obesity, *n* (%)	14 (16.2)	14 (16.2)	0.9
PAD, *n* (%)	17 (19.8)	16 (18.4)	0.8
Atrial fibrillation, *n* (%)	25 (29.1)	24 (27.6)	0.7
Previous PCI, *n* (%)	51 (59.3)	28 (32.2)	0.001
Previous CABG, *n* (%)	10 (11.6)	10 (11.6)	0.9
Previous MI, *n* (%)	43 (50.0)	23 (26.4)	0.001
Diagnosis			0.003
NSTEMI, *n* (%)	9 (10.5)	14 (16.1)	
Unstable Angina, *n* (%)	7 (8.1)	4 (4.6)	
STEMI, *n* (%)	6 (7.0)	17 (19.8)	
Chronic coronary syndrome, *n* (%)	29 (33.7)	13 (15.1)	
Acute heart failure, *n* (%)	1 (1.1)	6 (6.9)	
Chronic heart failure, *n* (%)	34 (39.5)	33 (38.4)	
Physiologic variables			
BMI, kg/m2 (mean ± SD)	26.1 ± 4.3	25.9 ± 4.1	0.5
LVEF, % (mean ± SD)	36.2 ± 7.6	35.0 ± 9.0	0.7
Mehran score, (mean ± SD)	6.4 ± 4.1	7.8 ± 5.3	0.08
Laboratory variables			
Creatinine admission, mg/dL (mean ± SD)	1.3 ± 0.5	1.37 ± 0.92	0.9
Hemoglobin admission, g/dL (mean ± SD)	13.3 ± 1.9	12.9 ± 2.2	0.12
HbA1c, mmol/mol (mean ± SD)	47.3 ± 11.2	50.0 ± 13.0	0.6
hsTnT peak, pg/mL (mean ± SD)	67.4 ± 302.4	125.0 ± 262.1	0.004
eGFR, mL/min (mean ± SD)	58.3 ± 25.5	58.0 ± 37.0	0.3
NT-proBNP, pg/mL (median, IQR)	1337, 597.5–3449.8	2463, 833.3–7276.0	0.13
Medications			
SGLT2-i			<0.001
Dapaglifozin 5 mg, *n* (%)	0	0	
Dapaglifozin 10 mg, *n* (%)	56 (65.1)	0	
Empaglifozin 10 mg, *n* (%)	17 (19.8)	0	
Empaglifozin 25 mg, *n* (%)	13 (15.1)	0	
RAAS-i			<0.001
None, *n* (%)	20 (23.3)	41 (47.7)	
ACE-i, *n* (%)	18 (20.9)	32 (37.2)	
ARBs, *n* (%)	19 (22.1)	11 (12.8)	
ARNI, *n* (%)	29 (33.7)	2 (3.3)	
Statin			<0.001
None, n (%)	14 (16.3)	47 (54.7)	
High-intensity statin, *n* (%)	44 (51.2)	21 (24.4)	
Low-intensity statin, *n* (%)	29 (33.7)	18 (20.9)	
Diuretics			<0.001
None, *n* (%)	27 (31.4)	48 (55.8)	
Loop diuretics, *n* (%)	19 (22.1)	23 (26.7)	
Loop diuretics + MRA, *n* (%)	16 (18.6)	5 (5.8)	
MRA, *n* (%)	24 (27.9)	8 (9.3)	
Thiazide, *n* (%)	0 (0)	2 (2.3)	
Other type 2 diabetes mellitus therapies			0.7
None, n (%)	47 (54.7)	46 (53.5)	
Metformin, n (%)	21 (24.4)	21 (24.4)	
Sulfonylureas, n (%)	0 (0)	0 (0)	
DPP-4 inhibitors, n (%)	1 (1.2)	1 (1.2)	
GLP-1 receptor agonist, n (%)	2 (2.3)	2 (2.3)	
Insulin, n (%)	9 (10.5)	10 (11.6)	
Metformin + insulin, n (%)	4 (4.7)	4 (4.7)	
Other, n (%)	2 (2.3)	2 (2.3)	
Procedural variables			
PCI, *n* (%)	36 (41.9)	53 (61.6)	0.015
Radial access, *n* (%)	65 (75.6)	39 (45.3)	0.001
Complex PCI, *n* (%)	12 (14.0)	19 (22.1)	0.2
IABP, *n* (%)	1 (1.2)	5 (5.8)	0.4
Contrast volume, mL (mean ± SD)	82.6 ± 53.8	118.0 ± 87.0	0.001
Creatinine peak post, mg/dL (mean ± SD)	1.2 ± 0.5	1.4 ± 1	0.7
Outcomes			
CI-AKI, *n* (%)	8 (9.3)	21 (26.7)	0.016
CRRT, *n* (%)	0 (0)	5 (5.8)	0.059
All cause-death, *n* (%)	3 (3.5)	18 (20.9)	<0.001

Continuous variables are presented as median (IQR) or mean ± SD, whereas categorical variables are presented as n (%). ACE-i, angiotensin-converting enzyme inhibitors; ARBs, angiotensin re-ceptor blockers; ARNI, Angiotensin Receptor Neprilysin Inhibitor; BMI, body mass index; CABG, Coronary Artery Bypass Graft surgery; CRRT, continuous renal replacement therapy; DPP-4 in-hibitors, dipeptidyl peptidase 4 inhibitors; eGFR, estimated glomerular filtration rate; HbA1c, he-moglobin A1c; hsTnT, high sensitivity troponin T; IABP, Intra-Aortic Balloon Pump; LVEF, left ventricle ejection fraction; MI, myocardial infarction; NSTEMI, non-ST-elevated myocardial in-farction; NT-proBNP, N-terminal pro-B-type natriuretic peptide; PAD, peripheral artery disease; PCI, percutaneous coronary intervention; RAAS-I, renin–angiotensin aldosterone system inhibitors; SGLT2-i, sodium–glucose cotransporter-2 inhibitors; STEMI, ST-elevated myocardial infarction.

**Table 3:** Odds ratios (OR) of primary endpoint of CI-AKI after invasive iodinate contrast medium procedure by univariable and multiple logistic regression analysis. eGFR, estimated glomerular filtration rate; LVEF, left ventricle ejection fraction; PCI, percutaneous coronary intervention; RAAS-i, renin–angiotensin aldosterone system inhibitors; SGLT2-i, sodium–glucose cotransporter-2 inhibitors; CI, confidence interval.

Characteristics	Univariable			Multivariable		
	OR	95% CI	*p*-Value	OR	95% CI	*p*-Value
Age	0.98	0.94, 1.01	0.2			
Gender	0.95	0.26, 2.78	0.9			
LVEF on admission	1.00	0.96, 1.05	0.9			
PCI	2.11	0.94, 5.01	0.076	1.33	0.44, 3.97	0.6
Contrast volume	1.00	1.00, 1.01	0.072	1.00	1.00, 1.01	0.6
SGLT2-i use	0.36	0.15, 0.82	0.019	0.41	0.16, 0.90	0.045
eGFR on admission	1.02	1.00, 1.03	0.016	1.02	1.00, 1.03	0.025
Mehran score	1.05	0.97, 1.14	0.2			
Diabetes	2.33	0.97, 4.68	0.4			
RAAS-i therapy	0.92	0.41, 2.14	0.8			

**Table 4:** Odds ratios (OR) of secondary composite endpoint of CRRT and all-cause mortality after invasive iodinate contrast medium procedure by univariable and multiple logistic regression analysis. eGFR, estimated glomerular filtration rate; LVEF, left ventricle ejection fraction; PCI, percutaneous coronary intervention; RAAS-i, renin–angiotensin aldosterone system inhibitors; SGLT2-i, sodium–glucose cotransporter-2 inhibitors; CI, confidence interval.

Characteristics	Univariable			Multivariable		
	OR	95% CI	*p*-Value	OR	95% CI	*p*-Value
Age	1.05	1.0, 1.10	0.056			
Gender	0.83	0.19, 2.68	0.8			
LVEF on admission	0.95	0.91, 1.00	0.063	1.01	0.95, 1.07	0.7
PCI	1.22	0.52, 2.92	0.6			
Contrast volume	1.00	1.00, 1.01	0.2			
SGLT2-i use	0.11	0.02, 0.32	<0.001	0.12	0.03, 0.40	0.002
eGFR on admission	0.95	0.92, 0.97	<0.001	0.95	0.92, 0.98	0.001
Mehran score	1.27	1.15, 1.43	<0.001	1.28	1.13, 1.47	<0.001
Diabetes	1.37	0.58, 3.32	0.5			
RAAS-i therapy	0.64	0.27, 1.55	0.3			

## Data Availability

Data are contained within the article.

## Supplementary Materials

The following supporting information can be downloaded at: https://www.mdpi.com/article/10.3390/jcm13072041/s1. Figure S1: Standardized mean differences in included covariates before and after propensity score matching, revealing improvements toward 0.0 (no difference) after the matching process; Figure S2: Jittered plot of matched and unmatched observations according to their distribution on propensity score values. The plot shows a very good overlap between matched treated and untreated patients, with no treated patients left after propensity score matching; Figure S3: Density of propensity score distribution in the treated and control groups before and after matching.
