# Supplementary material for: Contrast-Induced Acute Kidney Injury in Patients with Heart Failure on Sodium–Glucose Cotransporter-2 Inhibitors Undergoing Radiocontrast Agent Invasive Procedures: A Propensity-Matched Analysis
Source: J Clin Med. 2024 Apr 1;13(7):2041. doi: 10.3390/jcm13072041 (PMC11012317; doi:10.3390/jcm13072041)
Supplement: Supplementary file 1 [file jcm-13-02041-s001.zip › jcm-2897615-supplementary.pdf]

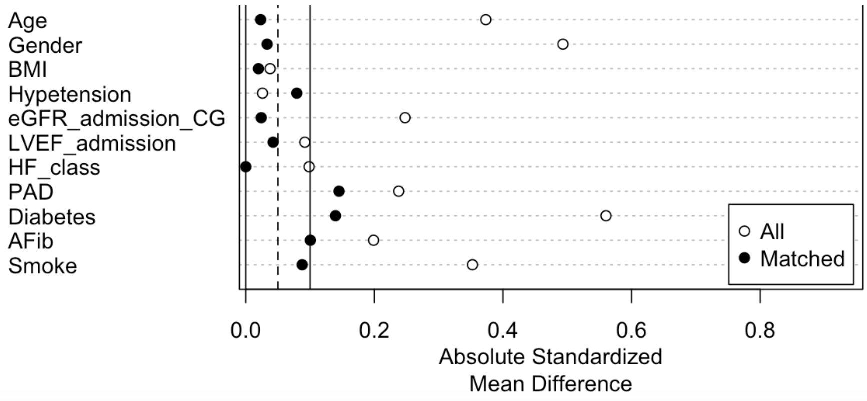

Figure S1. Standardized mean differences in included covariates before and after propensity score matching, revealing improvements toward 0.0 (no difference) after the matching process.

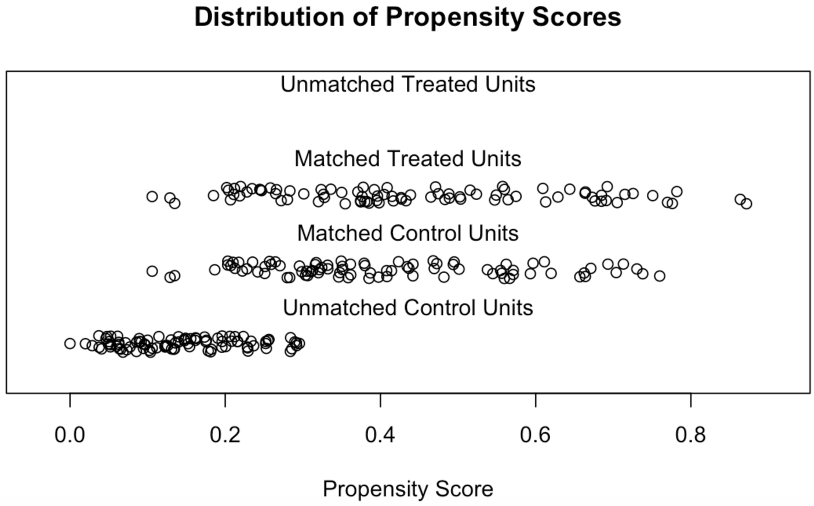

Figure S2. Jittered plot of matched and unmatched observations according to their distribution on propensity score values. The plot shows a very good overlap between matched treated and untreated patients, with no treated patients left after propensity score matching.

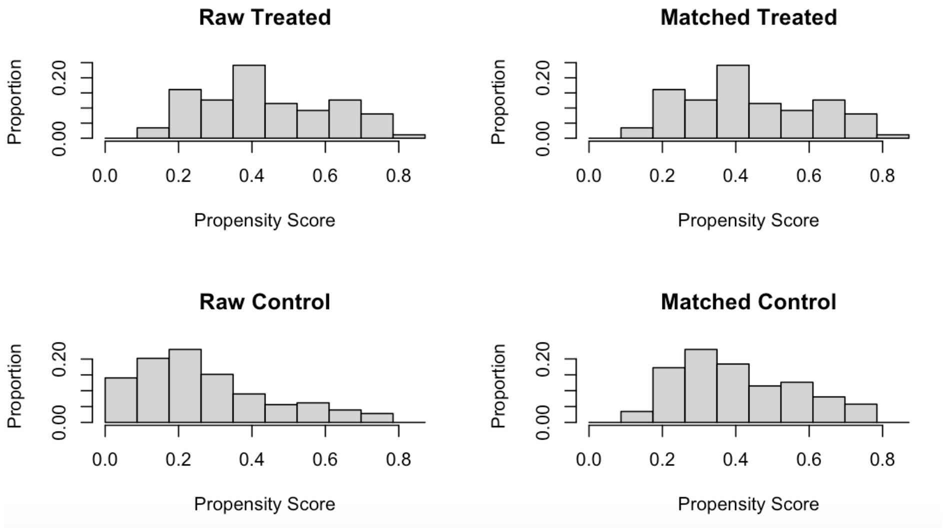

Figure S3. Density of propensity score distribution in the treated and control groups before and after matching.
